# Abstract of the Proceedings of the Buffalo Medical Association

**Published:** 1856-03

**Authors:** Sanford B. Hunt


ART. IV.—*Abstract of the Proceedings of the Buffalo Medical Association.*

TUESDAY EVENING, Feb. 5, 1856.

Owing to a failure in the distribution of notices, only Drs. White, Hawley, Rochester, Wyckoff, Baker, and Hunt were present. On motion of Dr. Hunt, Dr. White was called to the chair, and the meeting was adjourned to Tuesday, Feb. 12th, at the usual time and place.

TUESDAY EVENING, Feb. 12, 1856.

Association met.

Present—Drs. Strong, Mixer, Wilcox, Hawley, White, Rochester, Wyckoff, Baker, Hunt, Lay, and Howell.

Minutes of preceding meetings read and approved.

*Dr. Hawley* then read written reports of several cases in obstetric practice as follows:

*Rupture of the Uterus and Recovery.*

The following case, involving rupture of the uterus, is deemed of sufficient interest to be presented to the association:

Nov. 11, 1855, at 12½, at night. Patient a strong, rather fat, English woman, from 25 to 30 years of age, in her fifth labor, in charge of a midwife. Labor commenced at 7 o'clock in the evening, and the pains were severe from 9 o'clock. The head of the child, the face presenting anteriorly, was fully engaged in the pelvis; scalp much tumefied and the parts of the mother much swollen; the water, also, had been discharged. Pulse 81.

The patient stated that her previous labors had been short, the last occupying but two hours. She complained much of the severity of the pains, and expressed her fears that the child could not be delivered. There being nothing to distinguish it from other cases of severe labor, I determined to wait its progress. At about 2 o'clock, finding no progress made, I yielded to the desire of the patient and sent for forceps. Soon after, during the absence of the messenger, the patient complained of a severe and unusual pain at the epigastrium; this recurred at each successive throe. Not being able to relieve or account for this new symptom, I withdrew to an adjoining room, but was soon recalled to the bedside by a cry of anguish more intense than the preceding. The patient was alarmed, and declared herself suffering horrible pain at the epigastrium: considerable tympanitis existed at that place. Labor had entirely ceased, and a vaginal examination disclosed that the head of the child had receded and was lying loose in the pelvis, and a bloody fluid flowed from the passage. The motions of the child were for a short time violent, giving great pain to the mother, which she referred to the epigastric region. Vomiting of a dark offensive fluid occurred at this time; pulse 100. These symptoms I regarded as evidence of rupture of the uterus.

On the return of the messenger I immediately applied the forceps, and dispatched another messenger for counsel. I had brought the head fully into the lower strait, when one blade of the forceps warped, and the instrument slipped off and left the head to recede again. I now introduced the hand for the purpose of turning, but finding a membrane interposing between it and the feet, and believing them to have escaped from the uterus, abandoned the effort and awaited the arrival of Dr. White, at 4½, A. M. After an examination of the patient, it was deemed best to resort to the forceps again. The child was delivered at about 5 o'clock. Pulse 112.

Administered 1½ or 2 grains of opium shortly before the delivery, and the same quantity soon after, to be repeated every two hours. The patient was bandaged, and left with the strictest injunctions to keep quiet.

Nov. 12, 9½, A. M. Pulse 120; tympanitis extensive.

12½, P. M. Pulse 140; pain in the right hypochondrium, very severe, and extending to the spine.

5 o'clock, P. M. Pulse 132; pain diminished; tympanitis unaltered.

Applied bran poultice to the abdomen and continued the opium.

Nov. 13, 8½, A. M. Had slept at intervals during the night; is free from pain; other symptoms unchanged. During the preceding twenty-four hours she had taken 20 grains of opium, ice and ice water in small quantities frequently repeated, but no food.

Continue opium every three hours, and give chicken broth.

12½, P. M. Half an hour before a little chicken broth had been followed by vomiting, and at the time of my arrival she was suffering severe pains in the abdomen.

Discontinued the opium and gave 15 drops of laudanum, in vinegar, every hour, until the pain ceased, and then every two hours, and applied warm fomentations of hops over the abdomen.

5, P. M. Pain relieved; pulse 132; tympanitis diminished.

Continue the laudanum and the fomentations at intervals through the night; no food.

Nov. 14, 7, A. M. Pulse 120; tympanitis diminished.

Discontinue the opiate on account of nausea; crust coffee for nourishment.

2, P. M. Pulse 112; tympanitis and tenderness diminishing.

7, P. M. Pulse 100; has complained much of pain in the hypogastric region.

Gave an occasional powder of opium and applied fomentations.

Nov. 15, 8, A. M. Pulse 88; tympanitis and tenderness still diminishing; has suffered from pain since 12 at night.

5, P. M. Symptoms unaltered.

Prescribed cathartic.

Nov. 16, 9, A. M. Bowels have moved freely; pains much diminished; abdomen soft; pulse 92; some appetite and stomach tolerant of food; tenderness and some pain in the right hypochondriac and hypogastric regions.

℞ Laudanum gtt. x., every two hours.

5, P. M. Pulse 96; tongue furred; pain in hypogastrium.

Continue laudanum.

Nov. 17, 9½, A. M. Pulse 112; fur on the tongue increased; has suffered much pain all night.

Give 1¼ grs. opium every two hours, till pain is relieved; beef tea for nourishment.

From this time she improved steadily until the 21st, when she was dismissed from treatment. The urine was drawn off by the catheter during the whole illness. On the 20th of last month the menses returned, and the patient seemed restored to her usual health.

Nov. 15, 1855. Mrs. C. G——, a strong healthy young Irish woman in her third confinement, was delivered of a good sized male child after a natural labor of six hours. As I was leaving the house the patient complained of some uneasiness, which I supposed to be after pains, and for which an anodyne was prescribed. About two hours after I was aroused by the husband and informed that the patient was as bad as ever. I hastened to the house and was received with reproaches for having left my patient before she had completed her labor, the last throes of which she seemed to be suffering, and the attendants in instant expectation of the birth of a second child.

A vaginal examination discovered a tumor, measuring in its largest diameter, about three inches, and transversely about two inches, in the left wall of the vagina, just within the labia. The presence of this tumor excited in the patient a violent tenesmus, which strongly simulated labor pains, and which appeared to be a source of great suffering. A careful examination per vaginum, per anum, and by the eye, gave no satisfactory explanation of its nature, nor afforded any means of giving relief. In this dilemma I administered chloroform to assuage the sufferings of the patient, and sent for Dr. White in consultation. He having, by examination, nearly satisfied himself that we had a case of vaginal hernia to deal with, while in the act of further exploration, ruptured the walls of the tumor and dislodged a large coagulum of blood. The pains ceased instantly. The rupture of a vein and the consequent effusion of blood in the wall of the vagina, seems to have been the cause of this unusual and perplexing complication.

Dec. 23, 1855. Mrs. F——, a strong, healthy Irish woman, in her fifth confinement. Of the previous labors, two, in which the children were females, were easy and natural, and two in which the children were males, the positions were abnormal, and the deliveries instrumental. In this instance the face presented. The first three hours of labor sufficed fully to dilate the mouth of the uterus, but during the succeeding four hours no progress whatever was made, and the pains diminished in frequency and strength. Owing to disability of my left hand at the time, I sent for Dr. Eastman to apply the forceps. By their aid he had advanced the head considerably when the forceps slipped off and the head receded. Deeming it inexpedient to re-apply the instrument, I introduced my hand, brought down the feet, and delivered a living male child. *Query*—Does presentation of the face contra indicate the use of forceps?

*Prof. White* read the following report of a case of inversion of the uterus, with reposition on the eighth day:

### *Inversion of the Uterus.*

On Monday, 28th January, Dr. Storck called at my office requesting my attendance with himself and Dr. Dupré, upon a young female at No. 9, Huron street, who had been delivered of her first child upon the Tuesday previous.

Accompanying him, I found the patient, 19 years old, exsanguine, with quick pulse, and greatly exhausted from loss of blood. I found that she had been attended at the time of her delivery, by a German midwife, who stated that after a brief labor she had given birth to a male infant, weighing ten and one-half pounds. She also stated that the after-birth soon came away, accompanied by a large tumor, which descended into the vagina. This tumor she supposed to be a mole or false conception, and she stated that it was as "large as a common ball." The flooding at the time, she described as terrific, producing protracted syncope.

A day or two previous to my first visit, whilst making an effort to evacuate the bowels, the tumor had descended through the os externum and became suspended between the patient's thighs. All her efforts to remove the tumor proving unavailing, the midwife had sent for the gentlemen above-mentioned, for that purpose, and they now associated me with them in the treatment of the case.

The tumor was immediately recognized as the inverted uterus, as large as at the fourth month of pregnancy, which, with the external organs, was inflamed and tender. The uterus felt hard and inflexible, the body being apparently distended with blood from the ligated condition of the neck, and the parts were rendered exceedingly irritable by manipulations for the removal of the supposed false "conception or tumor."

By grasping the uterus gently and firmly with both hands, which it completely filled, compression was continued until the organ was relieved of its engorgement and considerably diminished in bulk. By continuing this firm but gentle compression, I was at length enabled to carry it up into the vagina. At this time the patient, having lost some blood during the effort, became very faint, in consequence of which, and the sensitiveness of the vulva, it was deemed prudent to omit farther efforts at exploration of the organ until the following morning.

Meanwhile the bowels were moved by enema, followed by an anodyne, and fomentations were applied to the abdomen and external genitals. The patient was also directed to take freely of broths and stimulants.

Tuesday, May 29, 12, M. Slept pretty well during the night; the hypogastric and vulval soreness is considerably diminished; the uterus lessened in size, and manifests some susceptability to indentation upon pressure.

The hæmorrhage has continued during the night; pulse 144, and feeble; has had a severe chill, and is greatly prostrated.

During the last three hours Dr. Storck has, at my suggestion, applied extract of belladonna to the neck of the uterus. At 12 o'clock placed the woman across the bed, her pelvis resting upon its edge and her feet being supported, one in the lap of Dr. Dupré the other in that of Dr. Storck. Prof. S. B. Hunt, who was kind enough to visit her, upon my invitation, gave her chloroform, so as gradually to bring her moderately under its influence. This effect was maintained throughout the succeeding operation.

I now placed myself upon my knees between the inferior extremities of the patient, a position admitting of free motion on my own part, giving complete control of the pelvis of the woman, and which could be maintained for a considerable period without unnecessary fatigue to the operator. Introducing the entire right hand into the vagina, the whole body and fundus of the organ were firmly and continuously compressed for some time. At length, keeping up the pressure, it was found, upon applying the thumb to the fundus, that a slight depression could be made. Having succeeded in dimpling the fundus, pressure was maintained with the thumb at that point until the hand became so fatigued as to be nearly powerless. To preserve this depression whilst the muscles of the hand were permitted to relax, a rectum bougie, about twelve inches in length and one in diameter, was carried along its palm fixed in the dimple, and pressure unintermittingly continued through it by the left hand outside the vulva. So soon as the intro-vaginal hand was sufficiently rested, pressure by it was recommenced and the bougie withdrawn.

Whenever these progressive efforts were resumed, the left hand was placed over the uterine tumor, which could now be distinctly felt in the hypogastrium. By means of the counter pressure above the pubis, a much greater degree of pressure could be made upon the depression in the fundus of the uterus without lacerating its vaginal connections. At length the fingers of the left hand being pressed well down into the abdomen, seemed to fasten upon or hook over the anterior uterine lip and aid in its reflexion over the organ. Thus securely held between the two hands, one within the vagina and the other upon the hypogastrium, these efforts at reduction were continued until I became nearly exhausted from fatigue. Gradually the concavity of the fundus was found to be deeper and deeper until it finally became completely restored. The bougie was now passed up to the fundus, penetrating twelve or more inches beyond the vulva, and gently maintained there by Prof. Hunt, whilst the patient was replaced in bed. My fingers were so benumbed by the long-continued unremitting pressure, as nearly to obliterate sensation, and at my request he also examined to ascertain whether the organs now occupied their normal relations. This being determined by him affirmatively, the bougie was gently withdrawn and the patient left with directions to give an anodyne, preserve quietude, and give her stimulants and nourishment freely. It may be added, that she seemed more comfortable than before the operation was undertaken, and expressed herself as feeling better than since her confinement. The hæmorrhage was, from this moment, completely arrested.

On the 30th, at 11, A. M., upon visiting her, with the same medical gentlemen who were present the day before, found her feeling better, with less pain and much more hopeful. The pulse had, however, but slightly diminished in frequency (140) or increased in force, and she still looked ex-sanguine.

Continued the treatment of the day previous, giving as much beef essence and brandy as the stomach will retain.

On Thursday, 31st, at 11, A. M., Dr. Storck informs me that the irritability of the stomach, which had been troublesome from her delivery, was now greatly increased, and it was with difficulty that she now retained the smallest quantity of fluid. The pulse is more feeble, and she is evidently sinking. Notwithstanding the free use of quinine and brandy, she expired at 5, P. M., on the same evening.

Feb. 1st, at 12, M., the *post-mortem* was made by Dr. Lemon, in the presence of Drs. Storck, Dupré, Hamestein, and Prof. Hunt, the last of whom, at my request, furnishes the following report of the condition of parts as they were found upon examination:

"The examination was held eighteen hours after death. Only the abdominal cavity was opened. All the tissues were extremely bloodless. The stomach and intestines were fully inflated with gas, but held hardly any fluid contents.

The walls of the intestines were white and translucent, and no trace of inflammatory injection could be found either upon them or any portion of the peritoneum. There was, however, a little serous effusion within the peritoneum, and between some of the convolutions of the intestine a very little lymph was exuded.

The uterus was dragged up and removed with as much of the vaginal canal as could be reached from within. Externally the uterus presented its normal shape and position, there being no trace of its recent dislocation. The vaginal mucous membrane and the os uteri, presented the dark color usual to the organ at this period after labor. The tissues were not softened, nor was there any laceration of them at any point.

Upon section through the posterior wall the same pale, bloodless appearance, noticed elsewhere, was presented. The uterine cavity contained a little altered blood. Upon washing the surface it presented no unusual appearance. The situation of the placenta was marked by the usual rough, flocculent surface.

The examination revealed no cause of death, unless the anæmic condition of the tissues may be considered as such. I have never before seen so bloodless a subject, with one exception; that of a girl who died from purpura hæmorrhagia. S. B. H."

The uterus and its appendages, were then submitted to the association for inspection.

This case is regarded as interesting in many respects. It will encourage the growing belief among accoucheurs, that reduction may be undertaken with reasonable hope of success, at a period much later than most writers have heretofore advised. Denman, Dewes, Velpeau, and others, believe any effort at restoration useless after a very few hours. In a valuable paper upon this subject from the editor of the Buffalo Medical Journal, to be found in the November number of that Journal for 1853, sixty-seven cases are collected, and all the facts pertaining to their reduction, so far as they could be obtained, are given. Most of the cases which were successfully treated were operated upon very soon after the accident. Thirty-two of the sixty-seven were not reduced, and a few "exceptional cases" at various periods after the first day. By this table Dr. Hunt has shown that treatment has, though very rarely, resulted in success at a later period than was formerly supposed practicable, and the above case furnishes another instance in support of the same position.

I have witnessed but three cases of inversion of the uterus. The history of one is given above. One of the others was seen and reduced soon after the accident; whilst the third was not visited until the fifteenth day — no effort at reduction being attempted. With my present views upon this subject I should abandon such a case as hopeless only after a prolonged effort at reposition. The accident occurred in 1842, and the female, then but 19 years old, now enjoys tolerable health, though the uterus still remains inverted in the vagina. The case is referred to, and the course of treatment pursued in part given by Prof. Hunt at page 335, in the paper already cited.

The position in which the patient was, in the present instance, placed for the operation, is deemed worthy of note. It will be perceived that it gives complete control of the pelvis, permits free motion of the person and arms of the operator, and may be maintained for a long time without fatigue. In this position he is able to render important assistance in the the most difficult stage of the operation, with the left hand over the hypogastrium. Nor was the use of the bougie unessential; by its assistance continuous pressure was maintained, whilst the hand was relieved for a short period, thus as it were tiring out the circular fibres of the neck. How much of the success of the operation was due to the relaxation of the neck from the application of the belladonna, if, indeed, any beneficial influence was exerted by it, I cannot determine. The moderate anæsthesia, continued during the efforts of manipulation, doubtless saved the patient much pain and lessened involuntary resistance. Whether the patient's chances of rallying were improved by the reposition, may, by some, be deemed doubtful. There were no lesions of the utero-vaginal connection found, indicating that such a degree of force had been used as to impair the integrity of, or excite inflammation in those tissues. The hæmorrhage which had been considerable during the previous twenty-four hours, ceased with reduction, and the woman was much more comfortable the day following than the one preceding. The patient doubtless died from loss of blood immediately attending the delivery of the placenta and inversion of the uterus, the disturbance of the system occasioned by the unnatural position of that organ during eight days, and the continuous drain by hæmorrhage during the same period. I believe it the opinion of all present that the shock of the operation of reposition was fully compensated by the increased comfort of the patient and arrest of flooding. She had, however, lost too large an amount of blood, reäction could not be established, though nature was aided in her efforts by all the resources of art.

*Prof. Rochester* then read a report of a case of poisoning by strychnia, treated successfully by camphor.

*Hospital Records.*

Feb. 2d, 1856. I. De F., aged 32 years, was admitted about 5, P. M. He had taken strychnia with a view of self-destruction. It was swallowed at 4, P. M., and was said by him to have been four grains in amount. Supposing a fatal effect certain and speedy, he mentioned to an acquaintance what he had done. Large draughts of whisky were poured down him immediately, and he was hurried to the hospital. In the absence of the house physician, Dr. B. H. Lemon, a messenger was dispatched for Dr. Rochester, who arrived at 7, P. M., and reports as follows:

The patient, a robust and athletic man, was much excited; his eye was bright and wild; his countenance flushed, and his respiration hurried; he complained of great thirst, and of a burning sensation at the epigastrium. The pulse was slightly accelerated, but not increased in force. He had been vomiting very freely; the emesis being produced by copious draughts of warm milk administered by the Sisters of Charity. The iris responded readily to the tests of sensibility. I was informed that he had had several tetanic convulsions, the last of which had just taken place, and was unusually long and severe. I directed a large sinapism to be applied to the epigastrium, and gave him 2 grs. of powdered camphor with half a teaspoonful of the tr. of camphor suspended in water. The sinapism had hardly been applied and the camphor taken, when a spasm commenced, first manifesting itself in the cervical muscles, then in those of the arms and chest, the latter producing slight opisthotonos, and lastly in those of the face, turning the eyes into their orbits and setting the lower jaw firmly. The countenance became turgid, and the jugulars were enormously distended. The pulse numbered 88 per minute, and preserved its rythm. Respiration seemed to be entirely suspended, no respiratory murmur was detected, but the heart's sounds were quite audible. The strong contraction of the pectoral muscles produced so much noise that the pulmonary auscultation was incomplete. The nares were distended, and remained so. The paroxysm lasted about three minutes, and then ceased with sudden muscular relaxation and with a deep inspiration. The Sister of Charity in attendance told me that the preceding convulsion had been longer, and that it was cut short by the application to the nares of the vapor of strong aqua ammonia. The spasm over the patient complained of slight headache and of intense thirst; his respiration was again hurried and his wild manner returned. His aspect and condition were not unlike those of Myer, the hydrophobic patient whom I saw with Dr. Hawley, and other medical gentlemen, some two years ago.

I directed the sinapisms to be removed from the epigastrium, and placed over the cervical and dorsal vertebræ, and repeated the camphor as before, with the addition of half a grain of morphine. At 7.35, a spasm similar to the one described, occurred; confident in the antidote properties of the camphor, I directed it to be given every fifteen minutes, and left the patient in charge of Dr. J. D. Freeman, who kindly consented to remain with him until the arrival of Dr. Lemon. The latter returned to the hospital about 9, P. M., and reports: Respiration 55 per minute; pulse 80. A spasm at 9 o'clock and 10 minutes, duration two minutes. There was opisthotonos, with intense contraction of masseter, sterno-mastoid, pectoral, biceps and gastrocnemii muscles. Spasms occurred at 9.25, 9.45, 10.15, and at 10.25. This proved to be the last, and was like a very severe chill; the one at 10.15 lasted five minutes and was very severe. At 11.30 there had been no recurrence of either spasm or chill. I directed a little chicken broth and weak brandy and water, and left him with directions to be called if there should be any untoward symptoms. His respiration at this time had fallen to 40; pulse 88. He vomited several times on taking the camphor, but the dose was immediately repeated and was not rejected on its second administration. To relieve the thirst and allay the vomiting, small pieces of ice were given.

Feb. 3d. Is much better this morning, has slept some; says he is hungry, is not thirsty, and has little or no pain at the epigastrium. Pulse 86; respiration 20; pupils natural; no headache.

Directed light diet and to remain in bed. The camphor, of which he took in all about ʒj, produced neither cerebral or gastric derangement.

Feb. 4th. Patient entirely recovered.

Dr. Rochester remarked that this was the second case reported by him to the society this year, where camphor had been successfully employed to counteract the effects of strychnia; he thought there was no doubt as to its properties as an antidote. Might it not possibly be successfully used in cases of traumatic and idiopathic tetanus?

*Dr. Baker*, from the committee on organization, reported a form for an act of incorporation, and moved that when the association adjourned it should adjourn to the evening of Wednesday, Feb. 20th. *Carried.*

STATE OF NEW YORK, } ss.

ERIE COUNTY, }

I. We, whose names are hereunto subscribed, do hereby, in pursuance of the several acts of the Legislature of the State of New York relating to the incorporation of *Benevolent*, *Charitable*, *Scientific* and *Missionary* Societies: *certify*, that we have associated, and do associate, for the purpose and object of establishing and maintaining an *association* in the city of Buffalo, in the county of Erie, and State of New York, under the name and title of "*The Buffalo Medical and Surgical Association.*"

II. That the association hereby formed, be known in law, by the name and title aforesaid.

III. That the particular business, object and purpose of said association shall be, to maintain, improve and advance the science of *Medicine* and *Surgery*, with their *collateral* sciences.

IV. That the affairs and business of said association shall be managed by its *Trustees.*

V. That the *President*, *Vice-president*, *Secretary*, *Treasurer*, and the *three members* of the *primary board*, shall be the Trustees thereof.

*Dr. Baker* proposed Dr. C. L. Dayton, for membership.
*Dr. Rochester* " Dr. B. H. Lemon, " " and
*Dr. Wilcox* " Prof. Geo. Hadley, " "

And the association then adjourned.

SANFORD B. HUNT, Secretary.

